# Synergistic Effects of Different Endophytic Actinobacteria Combined with Organic Fertilizer on Soil Nutrients and Microbial Diversity in *Camellia oleifera*

**DOI:** 10.3390/microorganisms13061396

**Published:** 2025-06-15

**Authors:** Yinghe Peng, Kunpeng Cui, Huimin Jian, Zhen Zhang, Longsheng Chen, Yanming Xu, Zhigang Li, Hongsheng Liu, Ting Xu, Rui Wang

**Affiliations:** 1 Hunan Academy of Forestry, Changsha 410004, China; pyhe240602894@163.com (Y.P.); csuftzz0526@163.com (Z.Z.); clongsheng@163.com (L.C.); xuyanming@hnlky.cn (Y.X.); lzg@hnlky.cn (Z.L.); 15802588171@163.com (H.L.); 2 Hunan Province Department of Forestry, The Forestry Affairs Center of Hunan Province, Changsha 410004, China; 3 Institute of Biomedical Engineering, College of Life Sciences, Qingdao University, Qingdao 266071, China; cuikunpeng@qdu.edu.cn; 4 School of Pharmacy and Bioengineering, Chongqing University of Technology, Chongqing 400054, China; hmjian@stu.cqut.edu.cn; 5 National Engineering Research Center for Oil-Tea Camellia, Changsha 410004, China; 6 Yuelushan Laboratory, Changsha 410004, China; 7 State Key Laboratory of Utilization of Woody Oil Resource, Changsha 410004, China

**Keywords:** *Camellia oleifera*, endophytic actinobacteria, organic fertilizer, soil physicochemical properties, microbial diversity

## Abstract

*Camellia oleifera*, a prominent species of edible oil tree in China, depends on improved soil fertility for its sustainable growth. Although the application of bacterial manure has been demonstrated to enhance soil nutrient conditions, the specific contributions of endophytes within fertilizers and their interactions with soil microbial ecosystems remain inadequately explored. This study investigates the impact of organic fertilizers combined with three endophytes (CoT10, CoH27, and CoH17) on the physicochemical properties, enzymatic activities, and microbial diversity of soils in *C. oleifera* plantations. Findings indicate that the integration of endophytes with organic fertilizers significantly improved soil nutrient levels (including total nitrogen, total phosphorus, and hydrolysable nitrogen), enzymatic activities (such as phosphatase, amylase, and nitrate reductase), and microbial diversity compared to the application of organic fertilizer alone. Notably, the endophyte CoT10, when applied alone with organic fertilizer, resulted in increased levels of total nitrogen, total phosphorus, and hydrolysable nitrogen in the soil, as well as a marked enhancement in the activities of soil phosphatase, amylase, and nitrate reductase. Furthermore, the combination of CoT10 with other endophytes in organic fertilizer improved the functionality of the other microorganisms and the efficiency of organic fertilizer utilization. This study underscores the synergistic effects of endophytes and organic fertilizers, providing scientific insights and practical strategies for the sustainable cultivation of *C. oleifera*.

## 1. Introduction

*Camellia oleifera*, a shrub or small tree classified within the Theaceae family, is a significant source of edible oil in China. This species is mainly distributed in the southern regions of China. The seeds of *C. oleifera* are highly esteemed for their capacity to produce tea oil, which is frequently referred to as the ‘Eastern olive oil’ due to its superior nutritional profile and associated health benefits, thereby rendering it an economically vital crop [1]. *C. oleifera* is mainly cultivated in subtropical hilly regions, encompassing approximately 4.5 million hectares in China. However, the yield of *C. oleifera* remains suboptimal, largely attributed to its substantial nutrient requirements. Currently, most cultivation occurs in red soil with low levels of nitrogen (N), phosphorus (P), and potassium (K) content, which has seriously restricted the potential for increased production of *C. oleifera*.

Proper fertilization practices have been recognized as a crucial agronomic strategy for optimizing the quality and yield of *C. oleifera* production. Nonetheless, the application of fertilizers is often conducted in an imprudent manner, with the inorganic fertilizers frequently utilized without appropriate adjustments to nutrient ratios tailored to the specific developmental needs of *C. oleifera*. This oversight not only hinders plant growth but also disrupts the soil ecosystem, adversely affecting its long-term fertility and health. A promising alternative involves the use of organic fertilizers as foundational amendments to improve soil conditions and enhance fertility [2]. However, challenges remain in the implementation of organic fertilizers, as the decomposition process can occasionally result in diminished nutrient availability. In extreme cases, improper application may lead to detrimental effects such as seedling scorching, root toxicity, and other growth-inhibiting consequences.

Endophytes, the symbiotic microorganisms that inhabit plant tissues, have been shown to exert various beneficial effects on plant growth and health [3]. Among these, endophytic actinobacteria have attracted considerable attention due to their capacity to produce a diverse array of bioactive secondary metabolites that can enhance plant vitality and resilience. Increasing evidence indicates that endophytes can positively influence the soil microbial ecosystem by facilitating the decomposition and transformation of organic matter, enhancing the availability of essential nutrients, and stimulating enzymatic activities within the soil. For instance, the endophyte *Phomopsis liquidambaris* has been shown to significantly increase microbial biomass and abundance in the rhizosphere of peanuts, enhance enzymatic activities, and improve soil fertility [4]. Similarly, dark septate endophytes (DSE) have been found to contribute to the soil microbial community structure in licorice plants, assist in the retention of soil moisture and organic matter, and enhance nutrient utilization, thereby promoting plant growth even under drought conditions [5]. In the context of *C. oleifera*, the endophytic actinobacteria CoT10 has exhibited the ability to enhance microbial diversity in rhizosphere soil, increase phosphorus and iron content, and ultimately support the growth of the host plant [6]. Additionally, incorporating microbial inoculants during the composting of organic fertilizer can expedite the transformation of organic matter, leading to faster compost maturation. When combined with organic fertilizers, endophytes can augment the microbial abundance in soil, facilitate nutrient uptake by *C. oleifera*, and enhance the efficiency of fertilizer utilization. Studies have indicated that integrating phosphate-solubilizing bacteria such as *Bacillus megaterium* and *Pseudomonas fluorescens* and nitrogen-fixing bacteria like *Azotobacter chroococcum* and *Azospirillum brasilense* with organic fertilizers can significantly enrich nutrient levels and enzymatic activities in carbon-deficient soils [7]. Despite these encouraging findings, there is a paucity of research specifically addressing the role of endophytic actinobacteria in *C. oleifera*, with limited studies focusing on their impact on phosphorus uptake and plant growth [6]. Moreover, investigations into the use of endophytes combined with organic fertilizers to improve the soil environment for *C. oleifera* remain relatively infrequent.

In previous studies, we successfully isolated multiple strains of endophytic actinobacteria from *C. oleifera*, specifically CoT10, CoH27, and CoH17, which exhibited notable growth-promoting effects on *C. oleifera* saplings [6,8]. Given these promising results, we hypothesized that the application of organic fertilizers enriched with these endophytes could influence microbial structure and function by improving soil fertility, thereby enhancing the growth of *C. oleifera*. Thus, the objective of this study is to investigate the effect of organic fertilizers containing various combinations of these endophytes on soil physicochemical properties, enzyme activity, and microbial diversity within mature *C. oleifera* plantations. The overarching goal of this study is to augment the availability of essential nutrients in plantation soils, thereby promoting the growth and yield of *C. oleifera*. By optimizing soil conditions and fostering nutrient-rich environments, this approach may offer a sustainable solution for enhancing crop productivity and resilience in *C. oleifera*.

## 2. Materials and Methods

### 2.1. Plant Materials and Microorganisms

This experiment was conducted on a plantation with six-year-old mature *Camellia oleifera* cv. ‘Xianglin No. 210’, located in the *C. oleifera* cultivation base in Ningxiang City, Changsha, Hunan Province, China. The soil physicochemical properties of the experimental site were as follows: total nitrogen (TN) 0.70 g·kg^−1^, total phosphorus (TP) 0.108 g·kg^−1^, total potassium (TK) 6.51 g·kg^−1^, hydrolysable nitrogen (HN) 53.19 g·kg^−1^, available phosphorus (AP) 0.3 mg·kg^−1^, available potassium (AK) 57.5 mg·kg^−1^, organic matter (OM) 7.45 mg·kg^−1^, and pH 4.55.

The organic fertilizer used in this experiment was a specialized *C. oleifera* fertilizer produced by Hunan ‘Lvlinhai’ Biotechnology Co., Ltd., Changsha, China. Its primary components included mushroom residue (20%), cow manure (60%), chicken manure (10%), and bovine bone meal (10%), with the total contents of N, P, and K at more than 5% and organic matter at more than 45%.

The strains used in this experiment were endophytic actinobacteria isolated from *C. oleifera* previously, specifically *Streptomyces* sp. CoT10, *Streptomyces* sp. CoH27, and *Streptomyces* sp. CoH17, each of which had shown promising plant growth-promoting effects in previous greenhouse trials. These endophytes were cultured in mannitol soybean agar (MS) medium at 30 °C for 7 days to ensure optimal growth and spore maturation. After the incubation period, mature spores were carefully collected from the surface of the culture media by gently scraping with sterile cotton swabs. The spores were then dissolved in sterile water to create a spore suspension. Using a hemocytometer, the concentration of this suspension was precisely adjusted to 1 × 10^8^ spore mL^−1^ for subsequent applications [9].

### 2.2. Experimental Design

In this experiment, nine treatment groups were established to investigate the effects of different combinations of endophytes and organic fertilizer on *C. oleifera*. The treatments included CoT10 combined with organic fertilizer (XL1); CoH27 combined with organic fertilizer (XL2); CoH17 combined with organic fertilizer (XL3); a combination of CoT10 and CoH27 with organic fertilizer (XL4); a combination of CoT10 and CoH17 with organic fertilizer (XL5); a combination of CoH27 and CoH17 with organic fertilizer (XL6); a mixture of CoT10, CoH27, and CoH17 with organic fertilizer (XL7); organic fertilizer without adding endophytic microorganisms as a control (XL8); and finally, a group without any treatment as negative control (XL9) (Table 1). Each treatment was carefully formulated to evaluate the potential synergistic or individual effects of these endophytes on soil improvement and plant growth.

Each treatment included 3 parallel groups, with each parallel group applied to 3 healthy and uniformly growing *C. oleifera* trees, resulting in a total of 9 trees per treatment. For each treatment, the endophyte-containing organic fertilizer was prepared by mixing 100 mL of endophytic actinobacteria spore suspension (1 × 10^8^ spores·mL^−1^) with 10 kg of organic fertilizer. The application of these fertilizer combinations occurred in March, using conventional fertilization methods, specifically through furrow application along the outer edge of the canopy projection area of the *C. oleifera* tree. Following a three-month period, in June, rhizosphere soil samples were collected from the 3 parallel trees and subsequently mixed to form a single group sample, resulting in 3 distinct groups for each treatment.

### 2.3. Determination of Chemical and Physical Properties of Rhizosphere Soil

Rhizosphere soil samples were collected from the experimental trees in each treatment. The soil property analysis included total nitrogen (TN), total phosphorus (TP), total potassium (TK), hydrolyzable nitrogen (HN), available phosphorus (AP), available potassium (AK), porosity (PV), organic matter (OM), pH, iron (Fe), calcium (Ca), and aluminum (Al) contents. The concentration of Fe, Ca, and Al were determined by atomic absorption spectrometry [10]. TN was measured via the Kjeldahl method [11]. TP was determined using the Mo-Sb colorimetric method [12]. TK was analyzed through flame photometric method [13]. HN content was determined according to Shi [14]. The ignition method was employed for assessing AP content [15]. AK was analyzed by neutral ammonium acetate extraction [16]. PV was determined using the ring knife technique. The ignition method was used to measure OM content [17]. Finally, soil pH was determined by a potentiometer in distilled water (1:2.5, *w*/*v*) [18].

### 2.4. Determination of Soil Enzyme Activity

Rhizosphere soil samples were carefully collected from the experimental trees in each treatment, with each sample placed in a separate sterile, sealed bag to prevent cross-contamination. Immediately following collection, the samples were quick-frozen using liquid nitrogen, then transported to the laboratory and stored at −20 °C for further analysis. Specific enzymes, including soil acid phosphatase (S-ACP), soil acid protease (S-ACPT), soil amylase (S-AL), soil nitrite reductase (S-NiR), soil nitrate reductase (S-NR), and soil urease (S-UE), were quantified. Each enzyme was analyzed following the protocols provided in the respective enzyme assay kits (Suzhou Keming Biotechnology Co., Ltd., Suzhou, China).

### 2.5. Diversity Analysis of Rhizosphere Soil Microorganisms

The stored rhizosphere soil samples at −20 °C were then sent to Allwegene Gene Technology Co., Ltd. in Beijing, China for microbial community analysis. Here, soil DNA extraction was performed using the PowerSoil DNA Isolation Kit (MoBio Laboratories, Carlsbad, CA, USA). The bacterial 16S rRNA gene was amplified using the primers 338F(ACTCCTACGGGAGGCAGCAG) and 806R (GGACTACHVGGGTWTCTAAT) to profile the bacterial community [19]. High-throughput sequencing was conducted on the Illumina Miseq PE250 platform, providing robust and comprehensive sequence data. The sequences were processed through steps of assembly, quality filtering, and demultiplexing to yield high-quality sequences, which were then clustered into operational taxonomic units (OTUs) for community analysis [20]. Based on OTU clustering, alpha diversity analysis and beta diversity analysis were performed to assess microbial diversity within and between samples. Additionally, the taxonomic annotation offered classification information at multiple taxonomic levels, enabling detailed analyses of microbial community composition and comparisons across different treatment groups. The raw reads obtained in this study were submitted to the National Center for Biotechnology Information (NCBI) under the accession number PRJNA1264782.

### 2.6. Statistical Analysis

All data are presented as mean ± standard error (SE). Statistical analysis of the experimental data was performed using SPSS software (version 22.0; IBM Inc., Chicago, IL, USA). To assess the differences among the various treatment groups, an analysis of variance (ANOVA) was conducted with the Tukey multiple-range test to identify specific differences between the groups. The results were considered statistically significant when the *p*-value was less than 0.05. All of the soil properties were standardized, and the microbial abundance data underwent Hellinger transformation prior to conducting the partial redundancy analysis (RDA). An environmental correlation heat map was generated based on the Spearman correlation coefficients (Spearman’s *p* > 0.1, *p* < 0.05).

## 3. Results

### 3.1. Effect on Soil Element Contents Under Camellia Oleifera Plantation

The cultivation of *Camellia oleifera* is significantly influenced by the application of organic fertilizers, which are essential for enhancing the soil physicochemical properties, particularly in the regulation of vital nutrients. The addition of beneficial microorganisms into the organic fertilizers can facilitates the decomposition and transformation processes, thereby improving the efficiency of nutrient release and uptake. This study sought to evaluate the potential benefits of integrating *C. oleifera* endophytes with organic fertilizers to improve nutrient utilization efficiency within the soil. Various combinations of endophytes and organic fertilizers were formulated and subsequently applied to *C. oleifera* plantations.

As shown in Table 2, all treatments incorporating fertilizers exhibited enhanced soil nutrient levels in comparison to the negative control (XL9), implying that the application of organic fertilizers positively improved soil conditions. Furthermore, nearly all treatments that included endophytes demonstrated improvements in soil nutrient status relative to the control group (XL8). Notably, the organic matter content was significantly elevated in XL1 and XL5, with increases of 85.40% and 74.82%, respectively. Analysis of soil pH revealed that, in contrast to XL8, the pH levels increased in XL1, XL2, XL3, and XL5, whereas a decrease in pH levels was observed in XL4, XL6, and XL7. These findings suggest that the synergistic application of organic fertilizer and endophytes could effectively enhance soil nutrient status and organic matter content, while also affecting soil pH levels.

In comparison to XL8, most treatments exhibited varying levels of improvement in OM, TN, TK, AP, and AK content. Notably, the TN content in XL1 increased by 148.10%, HN by 80.04%, and AK by 83.53%. Conversely, XL3 showed a 30.35% increase in TK content, while the TP content in XL5 rose by an impressive 225.71%, with XL7 showing a 199.60% increase in TP. Additional analysis revealed significant increases in TN content across the soils treated with XL1, XL2, XL3, and XL5, rising by 148.10%, 29.11%, 34.18%, and 48.10%, respectively (Table 2).

These findings indicate that organic fertilizers containing endophytes, specifically CoT10, CoH27, and CoH17, were effective in enhancing soil TP content, with CoT10 alone demonstrating the most significant effect. Soil TP measurements demonstrated that XL1, XL2, XL3, and XL5 exhibited markedly higher TP levels compared to XL8, with increases of 120%, 74.29%, 14.28%, and 225.71%, respectively. These findings indicated that CoT10, CoH27, and CoH17, when applied individually, could enhance soil TP content, with the combination of CoT10 and CoH17 achieving the most pronounced effects. Conversely, other combinations were found to be less effective. Regarding TK, soils treated with endophyte-containing organic fertilizers exhibited an increase in TK content relative to the control; however, these differences were not statistically significant. This implied that these treatments had a limited influence on soil TK levels, possibly due to the intricate interactions between endophytes and nutrient availability.

In addition to the macronutrients mentioned above, the application of endophyte-containing organic fertilizers led to notable enhancements in soil elements (Table 2). Specifically, the calcium (Ca) concentration in XL5 rose over fivefold, indicating a substantial improvement in the availability of this element. Likewise, aluminum (Al) concentration increased by 60.34%, suggesting that the endophyte-enriched organic fertilizers may have facilitated the mobilization or transformation of Al within the soil, potentially improving soil structure and enhancing nutrient uptake. Furthermore, in XL7, iron (Fe) concentration showed a modest increase of 8.16%. Although this increase is comparatively smaller than the changes observed for Ca and Al, it nonetheless highlights the influence of endophytes on influencing soil mineral content.

### 3.2. Effect on Soil Enzyme Activity Under Camellia Oleifera Plantation

**Figure 1:**
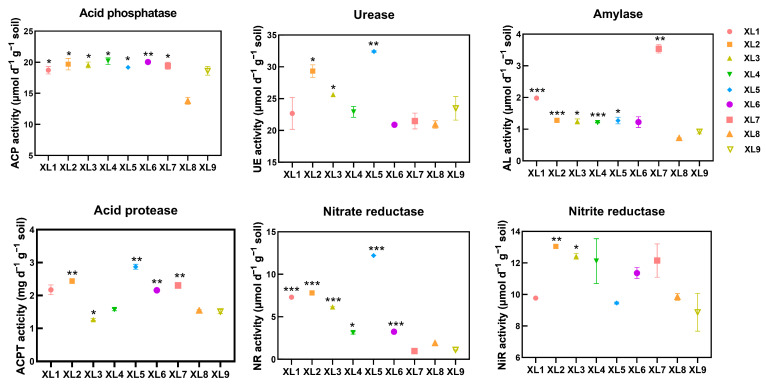
Soil enzyme activity in *Camellia oleifera* plantation with organic fertilizer with different endophytes. XL1: CoT10 + fertilizer; XL2: CoH27 + fertilizer; XL3: CoH17 + fertilizer; XL4: CoT10 + CoH27 + fertilizer; XL5: CoT10 + CoH17 + fertilizer; XL6: CoH27 + CoH17 + fertilizer; XL7: CoT10 + CoH27 + CoH17 + fertilizer; XL8: fertilizer only, control; XL9: without any treatment. Standard errors are shown (*n* = 3). Statistical comparisons with the control were made by the Tukey–Kramer test (* *p* < 0.05, ** *p* < 0.01, *** *p* < 0.001).

As illustrated in Figure 1 and Table 3, the application of endophyte-containing organic fertilizers led to a notable increase in soil enzyme activity when compared to the control (XL8). In particular, the activity of soil acid phosphatase (S-ACP) showed a significant increase across all treatments, with XL4 demonstrating the most pronounced rise of 36%. This indicates that endophyte-enriched fertilizers may enhance soil phosphatase activity, which is crucial for phosphorus availability. Soil urease (S-UE) activity was markedly elevated in XL2, XL3, and XL5, with XL5 showing the most substantial increase of 55%. This suggests that the strains CoT10 and CoH17 have a considerable effect on enhancing urease activity, potentially improving nitrogen availability through the decomposition of organic urea in the soil. Additionally, all treatments displayed increased soil amylase (S-AL) activity, with the combination of CoH27, CoH17, and CoT10 yielding an almost fourfold enhancement. Regarding soil acid protease (S-ACPT), XL2, XL5, XL6, and XL7 showed significant improvements compared to the control, with XL5 being the most effective, resulting in an 85.16% increase. Nitrite reductase (S-NiR) activity was significantly elevated in XL2 and XL3, with increases of 32.62% and 26.22%, respectively. This suggests that CoH27 and CoH17 contribute to the increased S-NiR activity. While most treatments showed improvements in nitrate reductase (S-NR) activity, XL7, which incorporated all three strains, exhibited a decrease in activity. This observation may indicate potential interactions or competition among the endophytes that affect their ability to enhance this specific enzyme activity.

### 3.3. Effect on Soil Bacterial Community Under Camellia Oleifera Plantation

**Figure 2:**
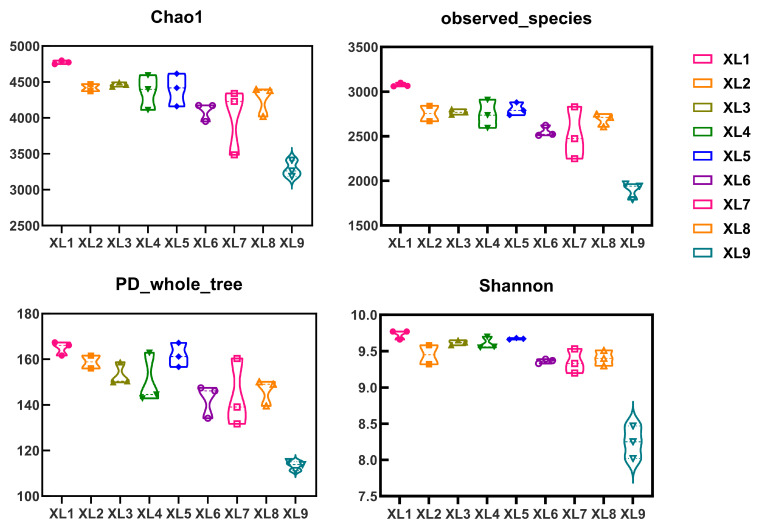
Changes in composition and diversity of rhizosphere bacterial community in *C. oleifera* under organic fertilizer with different endophytes. XL1: CoT10 + fertilizer; XL2: CoH27 + fertilizer; XL3: CoH17 + fertilizer; XL4: CoT10 + CoH27 + fertilizer; XL5: CoT10 + CoH17 + fertilizer; XL6: CoH27 + CoH17 + fertilizer; XL7: CoT10 + CoH27 + CoH17 + fertilizer; XL8: fertilizer only, control; XL9: without any treatment.

The soil microbial community was further sequenced and analyzed. The Statistics of splicing results are shown in Table A1. The results pertaining to OTUs revealed a hierarchical abundance of OTUs in the following order, XL1 > XL5 > XL4 > XL2 > XL3 > XL8 > XL6 > XL7 > XL9, with XL1 showing the highest species richness (Figure 2). This suggests that the application of CoT10 in conjunction with organic fertilizer (XL1) significantly enhances microbial species diversity, thereby promoting a more diverse microbial community within the rhizosphere. Further corroboration of these results is provided by the alpha diversity analysis utilizing the Chao1 index, which ranked as follows: XL1 > XL3 > XL5 > XL4 > XL2 > XL8 > XL7 > XL6 > XL9. This ranking confirms that the soils treated with CoT10-containing organic fertilizer (XL1) exhibited superior species richness compared to other treatments. Notably, the combinations represented by XL6 and XL7 were associated with a decline in microbial species richness, suggesting that these specific endophyte combinations may not effectively enhance microbial diversity. In terms of community diversity, the PD_whole_tree index yielded the following order: XL1 > XL5 > XL2 > XL3 > XL8 > XL6 > XL4 > XL7 > XL9. The XL1 treatment exhibited the highest community diversity, whereas the lowest was recorded in XL7. This observation further substantiates the notion that the integration of CoT10 with organic fertilizer fosters greater community diversity. Additionally, the Shannon index, another metric for assessing microbial diversity, reinforced these findings, indicating that XL1 displayed the highest diversity, while XL7 exhibited the lowest. Collectively, these results suggest that specific combinations of endophyte and organic fertilizers exert varying degrees of influence on microbial diversity than others, with XL1 (CoT10-containing fertilizer) emerging as the most effective in enhancing both species’ richness and community diversity in the rhizosphere soil of *C. oleifera* plantations.

**Figure 3:**
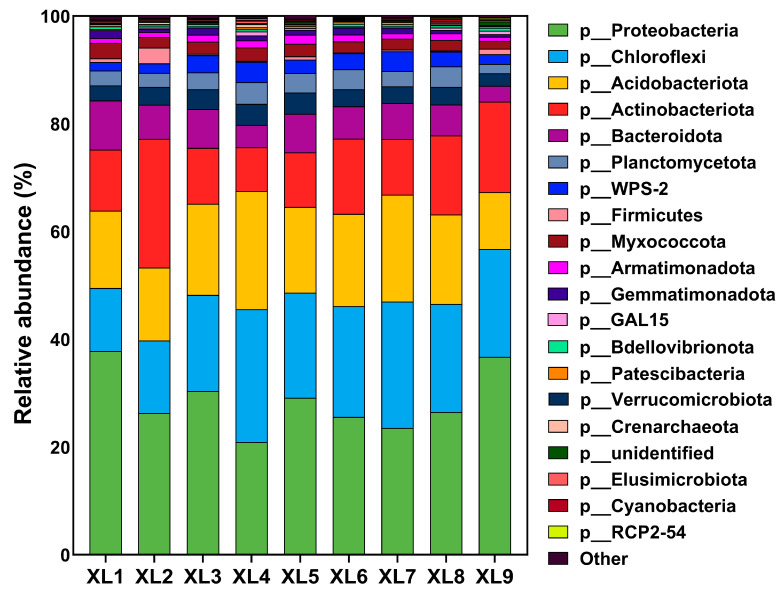
Relative abundance of dominant phyla in soil samples from different treatments in *C. oleifera* under organic fertilizer with different endophytes. XL1: CoT10 + fertilizers; XL2: CoH27 + fertilizer; XL3: CoH17 + fertilizer; XL4: CoT10 + CoH27 + fertilizer; XL5: CoT10 + CoH17 + fertilizer; XL6: CoH27 + CoH17 + fertilizer; XL7: CoT10 + CoH27 + CoH17 + fertilizer; XL8: fertilizer only, control; XL9: without any treatment.

To assess the structure of the microbial community in the rhizosphere of *C. oleifera* following the application of various organic fertilizer treatments containing endophytes, we conducted an analysis and annotation of the OTUs exhibiting 97% similarity. As illustrated in Figure 3, a total of 20 dominant phyla were identified. The predominant phyla across all samples included *Proteobacteria*, *Chloroflexi*, *Acidobacteriota*, *Actinobacteriota*, *Bacteroidota*, *Verrucomicrobiota*, *Planctomycetota*, *Firmicutes*, *Myxococcota*, *Armatimonadota*, *Gemmatimonadota*, *Bdellovibrionota*, *Patescibacteria*, *Crenarchaeota*, *Elusimicrobiota*, and *Cyanobacteria*. Comparative analysis of the treatments against the control group revealed notable alterations in microbial composition. In XL1, which incorporated the CoT10 strain, we observed an increase in the abundance of *Proteobacteria* alongside a decrease in *Chloroflexi*. This shift suggests that the application of CoT10 in organic fertilizers may favor the proliferation of *Proteobacteria*, a group recognized for its involvement in nitrogen cycling. In XL2, which included CoH27, there was a notable increase in the abundance of *Actinobacteriota*. The increase suggests that CoH27 may contribute to enhanced organic matter mineralization and nutrient cycling in the rhizosphere. In XL4 and XL7, which contained both CoT10 and CoH27, we noted an increase in the abundance of *Chloroflexi* and *Acidobacteriota*. The increased abundance of these phyla in XL4 and XL7 implies that the combination of CoT10 and CoH27 may stimulate microbial populations engaged in the decomposition of soil organic matter and nutrient cycling, thereby enhancing soil fertility and health. These results underscore the intricate interactions between endophytes and microbial communities in the rhizosphere, with specific strains fostering the growth of particular microbial phyla that contribute to improved soil quality and plant growth.

**Figure 4:**
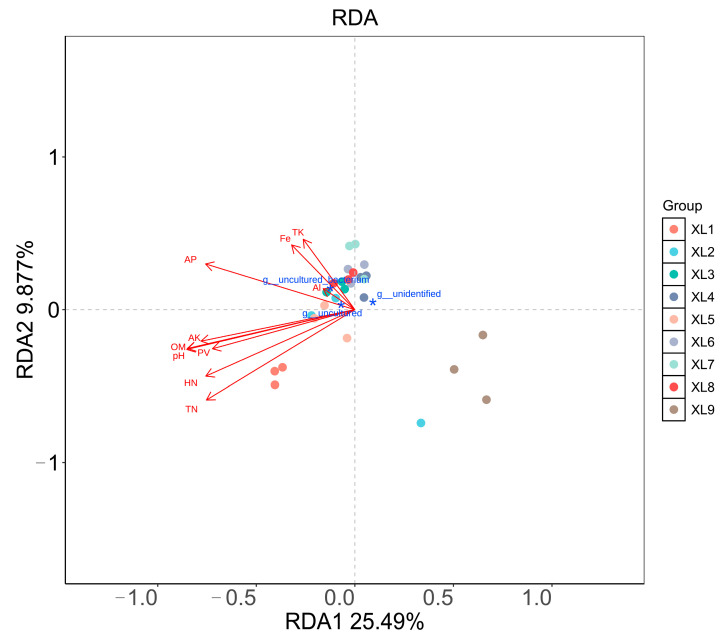
Redundancy analysis (RDA) for bacterial community (blue asterisk) and physiochemical properties (red arrows) of rhizosphere soil samples across different treatments in *C. oleifera* under organic fertilizer with endophytes. Physicochemical properties include soil pondus hydrogenii (pH), porosity (PV), soil organic matter (OM), total nitrogen (TN), total potassium (TK), hydrolyzable nitrogen (HN), available phosphorus (AP), available potassium (AK), iron (Fe), and aluminum (Al). XL1: CoT10 + fertilizer; XL2: CoH27 + fertilizer; XL3: CoH17 + fertilizer; XL4: CoT10 + CoH27 + fertilizer; XL5: CoT10 + CoH17 + fertilizer; XL6: CoH27 + CoH17 + fertilizer; XL7: CoT10 + CoH27 + CoH17 + fertilizer; XL8: fertilizer only, control; XL9: without any treatments.

The interaction between various environmental factors and microbial communities within the rhizosphere soil of *C. oleifera* was investigated using redundancy analysis (RDA). As shown in Figure 4, the first two axes of the RDA, RDA1 and RDA2, accounted for 25.49% and 9.877% of the variation, respectively. The results from the RDA indicate a significant difference in microbial community composition in the rhizosphere of *C. oleifera*, contingent upon the specific combinations of endophytes and organic fertilizers employed, with minimal variation observed within the treatment groups. Furthermore, the analysis highlights that soil parameters such as HN, TN, AK, pH, OM, and PV exerted a considerable influence on XL1 and XL5 treatments. This suggests that the application of CoT10 and the CoT10 + CoH17 combination effectively improved the soil environment, likely enhancing soil nutrient availability and conditions conducive to microbial habitation, which may ultimately promote healthier and more productive growth of *C. oleifera*.

**Figure 5:**
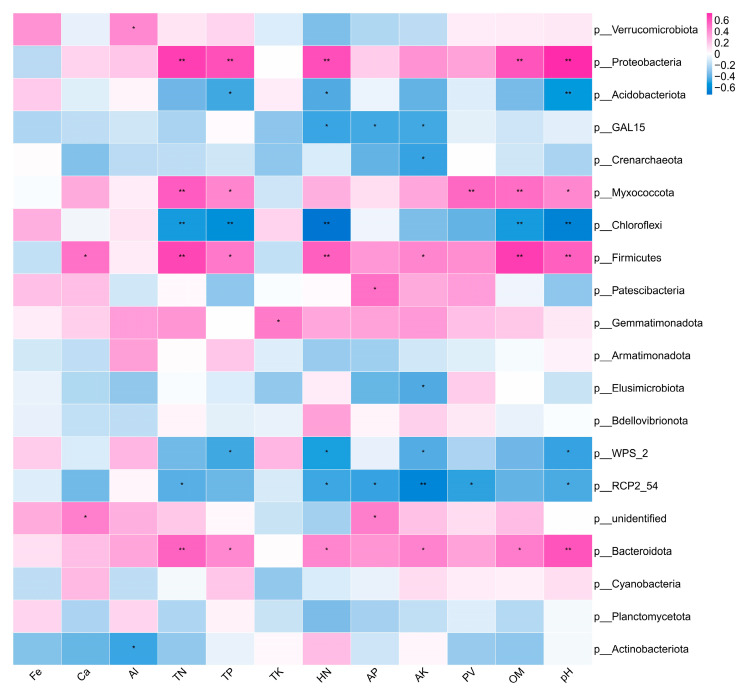
Species environmental factor-correlated heat map of rhizosphere soil samples across different treatments in *C. oleifera* under organic fertilizer with endophytes, where the X and Y axes are environmental factors and species, respectively, and the correlation coefficient R-value and corresponding *p*-value are obtained by calculation. R-values are shown in different colors in the figure, 0.01 < *p* ≤ 0.05 *, 0.001 < *p* ≤ 0.01 **. The upper legend is the color range of different R-values. Cluster analysis of species was performed, as shown in the heat map. Physicochemical properties include soil pondus hydrogenii (pH),porosity (PV), soil organic matter (OM), total nitrogen (TN), total phosphorus (TP), total potassium (TK), hydrolyzable nitrogen (HN), available phosphorus (AP), available potassium (AK), iron (Fe), calcium (Ca), and aluminum (Al).

We further selected the top 20 most abundant microbial phyla present in soil samples and subsequently calculated the correlation coefficients between these microbial groups and various environmental factors at the phylum level. The resulting correlation matrix was visualized as a heat map, as shown in Figure 5. This analysis encompassed 12 environmental factors: TN, TP, TK, HN, AP, AK, Fe, Ca, Al, PV, OM, and pH. Sixteen phyla exhibited significant correlations with the assessed environmental factors. For instance, *Proteobacteria*, *Myxococcota*, *Firmicutes*, and *Bacteroidota* showed significant positive correlations with TN, TP, and OM, among other factors. These microbial groups are likely to play a crucial role in nutrient cycling and organic matter processing within the soil, thereby contributing to soil health and the availability of nutrients for the growth of *C. oleifera*.

## 4. Discussion

The yield of *Camellia oleifera* is significantly influenced by the practices associated with fertilizer application [21]. Organic fertilizers not only provide essential nutrients for the growth of *C. oleifera* but also harbor beneficial microbial communities. The proliferation of these beneficial microbes in the soil contributes to improved soil quality, optimizes the growth environment for crops, and ultimately leads to enhanced crop yields [2]. The introduction of microorganisms can expedite the decomposition and transformation processes during the maturation of organic fertilizers, thereby increasing their fertilizer efficiency. Unlike rhizosphere-associated beneficial microbes, endophytes residing within the plant may exert a more direct and favorable impact on plant growth due to their specific ecological niches. In this study, we integrated previously isolated endophytic strains of *C. oleifera*, characterized by growth-promoting traits such as phosphate solubilization, nitrogen fixation, siderophore production, and IAA synthesis [8], into organic fertilizers. This approach aims to enhance the utilization efficiency of organic fertilizers, enrich essential soil nutrients, and ultimately increase the yield of *C. oleifera* plantations.

Numerous studies have shown a close relationship between soil nutrient content and microbial activity [2]. In the present study, the application of organic fertilizers enriched with endophytes was found to effectively improve soil physicochemical properties, elevate soil nutrient levels (specifically N, P, and K), and increase soil enzyme activity. These findings indicates that the addition of endophytes facilitated the decomposition and transformation of organic matter present in both the fertilizer and the soil. Consequently, these improvements contributed to a more conducive growth environment for *C. oleifera*.

Soil porosity, organic matter, and pH are critical factors that significantly influence the rhizosphere environment and, consequently, plant growth. Organic matter enhances the absorption of other essential nutrients, effectively contributing to nutrient retention within the soil [22]. In the present study, organic matter content increased in the treatments where endophytes were applied individually (XL1, XL2, and XL3). This indicates that the individual strains CoT10, CoH27, and CoH17 possess the ability to elevate soil organic matter levels. This result aligns with previous pot experiments [6], which also demonstrated that the application of single strain could improve soil organic content and nutrient availability. However, when endophytes were applied in combination, as seen in XL5 (CoT10 + CoH17), the increase in organic matter levels was less pronounced. This phenomenon may be attributed to potential nutrient competition or inhibitory interactions among CoT10, CoH27, and CoH17, which could diminish the efficacy of combined applications compared to those involving single strains. Additionally, it is plausible that these strains exhibit different interactions within the soil environment, leading to changes in microbial dynamics or enzyme activity that may hinder optimal organic matter accumulation.

*C. oleifera* is predominantly cultivated in nutrient-deficient red soils, characterized by low concentration of essential elements such as N, P, and K. These nutrients are crucial for plant growth and development: adequate N facilitates the synthesis of nitrogenous compounds, including proteins, enzymes, chlorophyll, and auxins, which stimulate plant growth; K plays a significant role in the interaction of various other nutrients within the plant and can even enhance N cycling; P, while the third most abundant nutrient after N and K, is often a limiting factor for crop yield, with approximately 40% of cultivated soils globally exhibiting P deficiency. Consequently, the application of phosphate fertilizer has become a key agronomic practice for improving crop yield and quality. Thus, hydrolyzable nitrogen, available phosphorus, and available potassium are the key indicators of soil fertility. In our study, XL1 notably increased soil hydrolyzable nitrogen, available phosphorus, and available potassium by 80.04%, 163.06%, and 83.53%, respectively (Table 2). Previous research have underscored the strong phosphate-solubilizing ability of CoT10, and our findings further reveal its efficacy in mobilizing soil nitrogen and potassium, demonstrating CoT10’s comprehensive ability to enhance soil nutrient levels. Furthermore, we observed that all endophyte-containing organic fertilizers increased soil available phosphorus, with CoH17 alone being the sole exception. However, when CoH17 was combined with CoT10, its effectiveness in increasing available phosphorus was notably enhanced. This suggests a synergistic interaction between CoT10 and CoH17, indicating their compatibility for combined application. The combination of all three strains together yielded the most pronounced effect, underscoring a synergistic effect when the three endophytes were utilized in conjunction with organic fertilizer, leading to a substantial increase in available phosphorus in the soil. Additionally, all treatments involving endophytes and organic fertilizer improved the available potassium content in the soil. Although none of the three strains showed potassium-solubilizing abilities in preliminary assessments, the incorporation of organic fertilizer likely facilitated the decomposition and transformation of potassium compounds, resulting in increased available potassium levels. Notably, CoH17 exhibited the least effect on increasing available potassium when applied alone, suggesting that it may be more advantageous to combine CoH17 with CoT10 rather than using it in isolation.

In addition to the three primary macronutrients (N, P, and K), soil elements such as iron (Fe), calcium (Ca), and aluminum (Al) are essential for plant growth, as they are involved in various physiological processes within the plant [23]. As shown in Table 2, soils treated with different endophyte-containing fertilizers exhibited increased concentrations of Fe, Ca, and Al compared to the control. This is particularly relevant in acidic red soils, where P is often bound to ions such as Ca^2^⁺, Fe^2^⁺, Fe^3^⁺, and Al^3^⁺, forming insoluble phosphates due to strong soil fixation, which restricts P availability for plant uptake. Previous studies have demonstrated that the three endophyte strains used in this experiment possess a robust capacity to solubilize Ca-P, Fe-P, and Al-P complexes, thereby converting bounded-P into a form accessible to plants. The current experiment corroborates these findings, as evidenced by the increased levels of Fe, Ca, and Al in the treated soils, indicating that the endophytes contributed to the dissolution of bound phosphates. Whether applied individually or in combination, the strains effectively dissolved Ca-P, Fe-P, and Al-P, thereby enhancing available P in the soil. Our results underscore the value of employing endophytes as a strategy to improve P bioavailability in nutrient-deficient or P-fixated soils, particularly in acidic conditions. These findings emphasize the comprehensive effects of endophyte-enriched organic fertilizers on not only macronutrients but also trace elements, which are essential for overall soil fertility and plant development.

Soil enzyme activity is a critical determinant of plant growth, as these enzymes function as natural biocatalysts that facilitate biochemical processes. Their activity is commonly used as an indicator of the intensity of soil biochemical processes and soil fertility [24]. Previous studies have shown that the application of organic fertilizers can enhance soil enzyme activity, including urease, phosphatase, and sucrase [25]. In this study, the application of endophyte and organic fertilizer treatment led to varying levels of increase in the activities of soil enzymes, such as phosphatase, urease, amylase, acid protease, nitrate reductase, and nitrite reductase. Acid phosphatase plays a significant role in the decomposition and mineralization of soil organic phosphorus, which can convert organic phosphorus compounds into inorganic phosphorus. Notably, phosphatase activity significantly increased in all treatments, indicating that the applied endophyte-containing fertilizers effectively boosted soil phosphatase activity. Urease activity serves as a crucial enzyme regulating soil N transformations and plays an indispensable role in nutrient cycling processes. The significant rise in urease activity in treatments involving CoT10 and CoH17 suggests increased nitrogen transformation, which subsequently elevated nitrogen availability for plant uptake. Amylase is responsible for breaking down starches into simpler sugars, thereby providing energy for soil microorganisms and promoting overall soil health. Acid protease aids in protein degradation in acidic environment, thereby enhancing nitrogen availability for plant uptake. Nitrite reductase is vital for the conversion of nitrite to ammonia, therefore facilitating nitrogen cycling and improving nitrogen availability in the soil. According to our results, the XL4 treatment achieved the highest phosphatase activity, while XL5 had the greatest increase in nitrate reductase, urease, and acid protease activities, and XL7 showed the highest amylase activity. This suggests that the combined applications of CoT10, CoH27, and CoH17 are more effective in enhancing soil enzyme activity, with CoT10 combined with either CoH27 or CoH17 being particularly optimal. Consistent with these results, the elevated levels of OM, TN, and AP in the soil were found in the combined application of CoT10 and CoH17 (XL5). This enhanced effect might be due to their ability to activate pertinent soil enzymes, thereby accelerating the decomposition and transformation of organic fertilizers, which in turn enhances nutrient availability.

The diversity of microbial communities within the rhizosphere is crucial for plant growth and soil fertility [26]. Organic fertilizers are considered effective in stabilizing microbial community within the rhizosphere and supporting its related functions [27]. To assess microbial community diversity and abundance, alpha diversity indices, including Chao1, PD_whole_tree, observed_species, and Shannon, are commonly used. Higher values of these indices signify increased species richness and community diversity. Specifically, the Chao1 and observed_species indices are indicative of species richness, whereas the Shannon and PD_whole_tree indices reflect community diversity, with higher values denoting greater microbial diversity [28]. Previous research has shown that organic fertilizers positively affect alpha diversity indices of soil microbial community, such as Shannon, Chao, and ACE, thereby promoting microbial reproduction and population expansion [29]. Similarly, previous research found that the introduction of CoT10 into the rhizosphere soil of *C. oleifera* resulted in increased soil microbial diversity and richness, enhancing microbial interactions and related functions. In this study, the incorporation of endophytes with organic fertilizer led to a significant improvement in soil microbial diversity indices in treatments with endophytic-enriched organic fertilizers compared to those with organic fertilizer alone. Notably, the CoT10 single-strain treatment achieved the highest increases across all indices, while the combined treatment of CoT10 and CoH17 (XL5) exhibited the highest diversity indices among the mixed treatments. This aligns with previous findings regarding nutrient elements and enzyme activity, indicating that CoT10 and CoH17 enhance microbial diversity and richness in the rhizosphere soil of *C. oleifera*. However, the diversity indices for XL6 and XL7 were linked to a reduction, suggesting a potential competitive or antagonistic interaction between CoH27 and CoH17. This interaction may have disrupted the micro-ecological balance in soil, leading to less favorable outcomes when applied together, thereby highlighting the importance of strain compatibility for maintaining optimal health of the rhizosphere microbiome. Consistent with this, the reduced abundance of certain beneficial taxa, such as *Proteobacteria* and *Actinobacteriota* (Figure 3), further confirmed the ecological imbalance in XL6 and XL7. Moreover, we noticed that the increases of soil porosity in XL6 and XL7 were not significant (Table 2), suggesting the limited the pore space might adversely affect microbial viability, thereby diminishing microbial diversity.

The increase in microbial abundance, particularly among critical functional groups such as *Proteobacteria*, *Acidobacteriota*, and *Actinobacteriota*, points to an enhancement in nutrient cycling capabilities within the soil ecosystem. These microbial taxa are acknowledged for their essential roles in nitrogen fixation, organic matter decomposition, and the overall maintenance of soil health [30]. The addition of CoT10 in XL1 resulted in a heightened abundance of *Proteobacteria*, while the addition of CoH27 in XL2 raised the abundance of *Actinobacteria*. Numerous studies have indicated that *Proteobacteria* are predominant in the rhizosphere microbiome, which aligns with their rapid growth rates [22]. This characteristic is consistent with the elevated OM content observed in XL1, where the carbon-rich nutrients in the soil are efficiently utilized by *Proteobacteria*, allowing for them to dominate within the microbial community. Additionally, *Proteobacteria* encompass various nitrogen-fixing bacteria and phosphate-solubilizing bacteria such as *Pseudomonas*, which corresponds with the increased TN and HN levels in the rhizosphere soil of XL1. *Actinobacteria* comprises numerous beneficial bacteria known for their ability to decompose organic matter and promote plant growth, including genera such as *Streptomyces, Frankia*, and *Nocardiopsis* [31,32]. Similarly, genera from the *Firmicutes* phylum, such as *Bacillus*, have been extensively studied for their roles in promoting nutrient uptake and plant growth [33]. The observed alterations in soil microbial community composition, coupled with the improvements in enzyme activity and nutrient levels, collectively indicate a synergistic relationship whereby endophyte-infused organic fertilizers modify the rhizosphere to promote nutrient availability. This enhancement likely creates a more conducive environment for *C. oleifera* growth, particularly by improving soil structure, nutrient retention, and microbial-driven nutrient cycling processes.

Overall, the addition of CoT10, either by itself or in combination with other strains, raised soil nutrient levels, increased soil enzymatic activity, and promoted beneficial microbial interactions, thereby improving the rhizosphere micro-ecological environment. As a consequence, these interactions facilitated the cycling and absorption of essential nutrients, supporting soil health and general plant growth. The long-term effects of endophyte-containing fertilizers on soil fertility and microbial community composition should be investigated further, and future research on the vigor and yield of *C. oleifera* plants should be conducted.

## 5. Conclusions

This study investigated the impact of organic fertilizers containing beneficial endophytes derived from *Camellia oleifera* on the soil environment supporting its growth. The findings demonstrated that organic fertilizer, when supplemented with specific endophytes, notably improved the physicochemical characteristics, enzyme activity, and microbial diversity within the rhizosphere soil. Specifically, fertilizers containing CoT10, whether applied alone or in combination with other endophytes, substantially enhanced soil fertility, indicating higher efficiency in nutrient delivery and utilization. This study recommends the practical application of endophyte-enriched fertilizers to improve fertilizer efficiency while reducing reliance on additional fertilizers and pesticides. Such research could provide valuable perspectives for establishing sustainable and eco-friendly farming practices that boost crop productivity.

## Figures and Tables

**Table 1:** Treatment groups of endophytic microorganisms and organic fertilizer.

Treatments	Groups
XL1	CoT10 + organic fertilizer
XL2	CoH27 + organic fertilizer
XL3	CoH17 + organic fertilizer
XL4	CoT10 + CoH27 + organic fertilizer
XL5	CoT10 + CoH17 + organic fertilizer
XL6	CoH27 + CoH17 + organic fertilizer
XL7	CoT10 + CoH27 + CoH17 + organic fertilizer
XL8	Control; organic fertilizer
XL9	Negative control; without treatment

**Table 2:** Effect of organic fertilizer with different endophytes on rhizosphere soil properties under *Camellia oleifera* plantation.

	XL1	XL2	XL3	XL4	XL5	XL6	XL7	XL8	XL9
pH	5.45 ± 0.25	5.15 ± 0.05	5.10 ± 0.00	4.85 ± 0.05	5.54 ± 0.05 *	4.85 ± 0.05	4.85 ± 0.05	5.00 ± 0.00	4.55 ± 0.00
Porosity (%)	71.54 ± 1.39	68.61 ± 1.43	64.38 ± 2.02	68.06 ± 4.26	66.69 ± 1.38	64.84 ± 0.72	65.38 ± 0.57	62.80 ± 1.51	61.14 ± 1.48
Organic matter (g kg^−1^)	25.4 ± 1.60 *	21.15 ± 0.35	20.9 ± 0.30	17.25 ± 1.35	23.95 ± 0.65 *	13.75 ± 0.75	15.3 ± 0.30	13.7 ± 1.90	7.45 ± 0.06
Total nitrogen (g kg^−1^)	1.96 ± 0.03 **	1.02 ± 0.01 *	1.06 ± 0.01 *	0.93 ± 0.03	1.17 ± 0.01 *	0.84 ± 0.02	0.85 ± 0.04	0.79 ± 0.05	0.70 ± 0.04
Total phosphorus (g kg^−1^)	0.77 ± 0.07 *	0.61 ± 0.03 *	0.40 ± 0.002	0.34 ± 0.01	1.14 ± 0.02 ***	0.33 ± 0.01	0.28 ± 0.01	0.35 ± 0.01	0.11 ± 0.01
Total potassium (g kg^−1^)	6.96 ± 0.32	6.91 ± 0.12	8.89 ± 0.47	6.91 ± 0.29	7.06 ± 1.10	7.90 ± 0.61	8.12 ± 0.68	6.82 ± 0.67	6.51 ± 0.72
Hydrolyzable nitrogen (mg kg^−1^)	137.37 ± 1.65 **	100.69 ± 6.42	97.21 ± 1.84	74.83 ± 1.47	78.87 ± 20.18	82.53 ± 1.83	75.38 ± 3.49	76.30 ± 5.87	53.19 ± 1.84
Available phosphorus (mg kg^−1^)	85.1 ± 6.60 *	73.55 ± 1.05 **	54.65 ± 4.65	63.75 ± 2.05 *	84.25 ± 0.45 **	78.49 ± 0.49 **	96.92 ± 1.62 **	32.35 ± 3.55	0.30 ± 0.00
Available potassium (mg kg^−1^)	161.14 ± 3.60 **	127.57 ± 0.73 **	90.97 ± 0.75	93.97 ± 6.17	153.29 ± 0.67 **	140.88 ± 0.80 **	100.58 ± 0.44 *	87.8 ± 2.71	57.50 ± 4.18
Iron (g kg^−1^)	31.55 ± 0.95	32.35 ± 1.55	32.6 ± 0.90	32.85 ± 3.75	31.45 ± 2.15	32.3 ± 2.50	33.15 ± 0.45	30.65 ± 2.05	29.85 ± 2.95
Calcium (g kg^−1^)	1.05 ± 0.21	0.60 ± 0.20	0.32 ± 0.40	0.70 ± 0.04 **	1.14 ± 0.30	0.29 ± 0.08	1.04 ± 0.05 **	0.18 ± 0.03	0.05 ± 0.00
Aluminum (g kg^−1^)	9.53 ± 1.77	9.09 ± 1.05 *	12.3 ± 1.10	9.87 ± 2.33	13.1 ± 0.20 **	9.6 ± 0.60	10.53 ± 1.08	8.17 ± 0.10	9.62 ± 0.49

Note: XL1: CoT10 + fertilizer; XL2: CoH27 + fertilizer; XL3: CoH17 + fertilizer; XL4: CoT10 + CoH27 + fertilizer; XL5: CoT10 + CoH17 + fertilizer; XL6: CoH27 + CoH17 + fertilizer; XL7: CoT10 + CoH27 + CoH17 + fertilizer; XL8: fertilizer only, control; XL9: without any treatment. Data are means ± SE (*n* = 3). Statistical comparisons with the control were made by the Tukey–Kramer test (* *p* < 0.05, ** *p* < 0.01, *** *p* < 0.001).

**Table 3:** Effect of organic fertilizer with different endophytes on soil enzyme activity under *Camellia oleifera* plantation.

	XL1	XL2	XL3	XL4	XL5	XL6	XL7	XL8	XL9
Acid Phosphatase	18.71 ± 0.59 *	19.69 ± 0.93 *	19.56 ± 0.47 *	20.18 ± 0.52 *	19.17 ± 0.04 *	20.03 ± 0.24 **	19.42 ± 0.58 *	13.79 ± 0.56	18.61 ± 0.69
Urease	22.67 ± 2.49	29.32 ± 0.98 *	25.65 ± 0.20 *	22.92 ± 0.85	32.41 ± 0.13 **	20.89 ± 0.13	21.48 ± 1.25	20.93 ± 0.59	23.48 ± 1.85
Amylase	1.98 ± 0.01 ***	1.28 ± 0.004 ***	1.24 ± 0.08 *	1.20 ± 0.01 ***	1.27 ± 0.10 *	1.22 ± 0.17	3.54 ± 0.13 **	0.72 ± 0.01	0.92 ± 0.07
Acid Protease	2.17 ± 0.15	2.44 ± 0.78 **	1.27 ± 0.03 *	1.57 ± 0.03	2.87 ± 0.08 **	2.16 ± 0.04 **	2.30 ± 0.03 **	1.55 ± 0.02	1.51 ± 0.06
Nitrate Reductase	7.30 ± 0.01 ***	7.81 ± 0.03 ***	6.17 ± 0.01 ***	3.13 ± 0.17 *	12.20 ± 0.02 ***	3.25 ± 0.002 ***	0.97 ± 0.004	1.90 ± 0.001	1.10 ± 0.02
Nitrite Reductase	9.77 ± 0.07	13.05 ± 0.03 **	12.42 ± 0.19 *	12.11 ± 1.42	9.46 ± 0.06	11.36 ± 0.35	12.15 ± 1.05	9.84 ± 0.22	8.87 ± 1.20

Note: XL1: CoT10 + fertilizer; XL2: CoH27 + fertilizer; XL3: CoH17 + fertilizer; XL4: CoT10 + CoH27 + fertilizer; XL5: CoT10 + CoH17 + fertilizer; XL6: CoH27 + CoH17 + fertilizer; XL7: CoT10 + CoH27 + CoH17 + fertilizer; XL8: fertilizer only, control; XL9: without any treatment. Data are means ± SE (*n* = 3). Statistical comparisons with the control were made by the Tukey–Kramer test (* *p* < 0.05, ** *p* < 0.01, *** *p* < 0.001).

## Data Availability

The original contributions presented in this study are included in the article. Further inquiries can be directed to the corresponding authors.

## Appendix A

**Table A1:** Statistics of splicing results.

Sample	Raw Tags	Clean Tags
XL1_1	69,172	64,941
XL1_2	48,414	45,946
XL1_3	45,680	43,251
XL2_1	68,448	66,361
XL2_2	24,941	23,993
XL2_3	48,305	46,741
XL3_1	97,867	94,393
XL3_2	49,043	47,354
XL3_3	81,099	77,621
XL4_1	35,828	34,610
XL4_2	59,179	57,519
XL4_3	64,131	62,621
XL5_1	49,134	47,661
XL5_2	117,833	113,444
XL5_3	282,653	273,342
XL6_1	166,279	160,931
XL6_2	237,655	230,963
XL6_3	131,795	125,677
XL7_1	209,166	202,892
XL7_2	26,211	24,076
XL7_3	365,223	356,826
XL8_1	81,657	79,783
XL8_2	96,888	93,374
XL8_3	69,074	67,290
XL9_1	55,083	53,306
XL9_2	32,538	32,247
XL9_3	43,940	42,819
